# A case of metastatic renal cell carcinoma and bile duct carcinoma treated with a combination of sunitinib and gemcitabine

**DOI:** 10.1186/s12885-015-1443-2

**Published:** 2015-05-22

**Authors:** Kotoe Takayoshi, Kosuke Sagara, Keita Uchino, Hitoshi Kusaba, Naotaka Sakamoto, Atsushi Iguchi, Eishi Baba

**Affiliations:** 1 Department of Medical Oncology, Clinical Research Institute, National Hospital Organization Kyushu Medical Center, 1-8-1 Jigyouhama, Chuo-ku, Fukuoka, 810-8563 Japan; 2 Department of Medicine and Biosystemic Science, Kyushu University Graduate School of Medical Sciences, Fukuoka, Japan; 3 Department of Urology, National Hospital Organization Kyushu Medical Center, Fukuoka, Japan; 4 Department of Comprehensive Clinical Oncology, Faculty of Medical Sciences, Kyushu University, Fukuoka, Japan

**Keywords:** ABCB1, Adverse event, Bile duct carcinoma, Gemcitabine, Plasma concentration, Renal cell carcinoma, Sunitinib

## Abstract

**Background:**

Metastatic renal cell carcinoma (mRCC) had been a chemo-refractory disease, but recent advances in multiple kinase inhibitors such as sunitinib have dramatically changed the clinical course of mRCC. Sunitinib is used for mRCC chemotherapy based on the favorable results of a recent clinical trial, but specific biomarkers predicting efficacy and safety are not yet available. Locally advanced bile duct carcinoma (BDC) has generally been treated with single agent gemcitabine or as doublet therapy with cisplatin. Concomitant occurrence of mRCC and BDC is extremely rare, and a standard therapeutic strategy has not been established.

**Case presentation:**

A 65-year-old woman was diagnosed as having multiple mRCC and intercurrent, locally advanced BDC. A single course of combination therapy with sunitinib (25 mg/day, day2-15) and gemcitabine (750 mg/m^2^, days 1, 8) was administered, and this showed obvious effects, with partial response for mRCC and stable disease for BDC. However, the patient also experienced severe adverse events, including hematological and various non-hematological toxicities; the combination therapy was then terminated on day 13 after its initiation. She recovered on day 28 and is alive 3.5 years after the diagnosis. The plasma trough levels of sunitinib and its active metabolite SU12662 on day 13 were 91.5 ng/mL and 19.2 ng/mL, respectively, which were relatively higher than in previous reports. Analysis of her single nucleotide polymorphisms (SNPs) detected TC in ABCB1 3435C/T, TC in 1236C/T and TT in 2677G/T, suggesting a possible TTT haplotype.

**Conclusion:**

A rare case of double cancer of mRCC and BDC was treated by combination chemotherapy. Although unknown synergistic mechanisms of these agents may be involved, severe toxicities might be possibly associated with high sunitinib exposure. Further exploration of combination therapy with sunitinib and gemcitabine is required.

## Background

Renal cell carcinoma (RCC) is one of the most serious urological malignancies. mRCC is initially diagnosed in 30 % of RCC patients, and 20–40 % of curatively operated RCC patients recur. Recently, new classes of molecular targeted agents, such as tyrosine kinase inhibitors and mTOR inhibitors, have become widely used for mRCC. Sunitinib is an oral tyrosine kinase inhibitor that targets vascular endothelial growth factor receptor (VEGFR)-1, −2 and −3, platelet-derived growth factor receptor (PDGFR)-α and -β, RET, and c-Kit. It has often been used for mRCC chemotherapy based on the favorable results of a phase III clinical trial showing superiority over interferon alpha [1]. Recent studies, however, have reported some adverse events including fatigue, bone marrow suppression, hand-foot syndrome, stomatitis, hypertension and hypothyroidism [1]. In a pivotal study of sunitinib, 38 % of the patients in the sunitinib group required dose interruptions due to adverse events, and 32 % required dose reductions to continue treatment courses [1]. Identifying biomarkers that can predict the response and adverse events of sunitinib is urgently needed in order to obtain the optimal effects of this drug.

Biliary tract cancer is rare in the Western countries, while it is relatively common in Latin America and Asia, including in Japan [2], and 50–90 % of patients was diagnosed as having advanced cancer and had a poor prognosis [3]. Combination chemotherapy consisting of fluoropyrimidine and gemcitabine has been given not only for metastatic biliary tract cancer but also for locally advanced disease. A recent clinical study showed the efficacy of the combination of gemcitabine and platinum for metastatic biliary tract cancer [4, 5]. While adverse events of gemcitabine such as myelosuppression, liver dysfunction, general fatigue, alopecia, and nausea were often observed, they were mostly tolerable in the pivotal clinical studies.

Concurrent occurrence of RCC and BDC is extremely rare. Only two cases have been reported in the literature, and the biological background of the synchronous primary malignancy was not clarified [6, 7]. Standard therapeutic strategies have generally not been established for cases of unresectable double primary cancers, and no chemotherapy was given to the above two cases. In the present case with concurrent mRCC and BDC, combination therapy of sunitinib and gemcitabine, which are both effective agents for each disease, was used, and both response and various adverse events were seen. Plasma concentrations of sunitinib and SU12662 were measured to assess the clinical effects induced by the combination therapy. Polymorphisms of specific genes encoding for metabolizing enzymes, efflux transporters, and drug targets involved in the pharmacokinetics (PK) and pharmacodynamics (PD) of sunitinib were also examined.

## Case presentation

### Case report

**Fig. 1:**
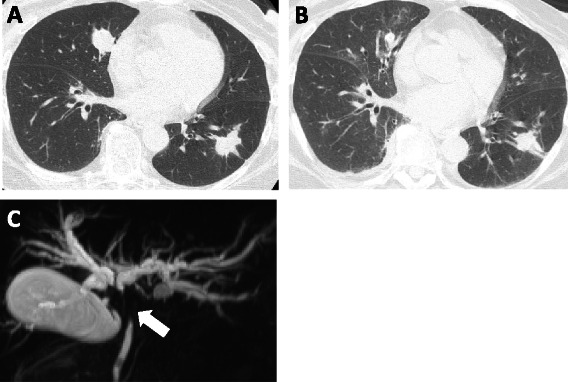
Images in clinical course: **a** Chest CT scan of lung metastases before introduction of sunitinib and gemcitabine. **b** Chest CT scan of lung metastases on day 20 after the administration. **c** Magnetic resonance cholangiopancreatography image demonstrated narrowing from upper common bile duct to bilateral intrahepatic bile ducts (arrow)

A 65-year-old woman was diagnosed with clear cell RCC in June 1998 and underwent radical left nephrectomy (pT2N0M0). Her disease status was good risk by Memorial Sloan Kettering Cancer Center criteria, and she was followed closely without therapy after the surgery. In December 2003, computed tomography (CT) showed multiple lung metastases. Interferon alfa-2a and sorafenib were administered sequentially. In August 2011, the tumor eventually progressed (Fig. 1a), and serum bilirubin and liver enzymes increased rapidly. Endoscopic retrograde cholangiopancreatography and magnetic resonance cholangiopancreatography examinations revealed narrowing with an irregular intraductal lumen of the area from the upper common bile duct to bilateral intrahepatic bile ducts (Fig. 1c). Cholangioscopy showed that the luminal mucosa was circumferentially narrowed with an irregular reddish surface. These findings were not likely to be metastatic RCC. She was diagnosed with upper BDC (T2N1M0). Endoscopic placement of a biliary stent immediately improved the bilirubin and liver enzyme levels in two days. Surgical resection of her BDC for the purpose of local control was not suitable because of RCC metastases. Because both cancers had a strong effect on her prognosis, treatment for both diseases was considered necessary. Thus, treatment consisting of 21-day cycles of sunitinib (25 mg/day; days 2–15 on, days 1 and 16–21 off) and gemcitabine (750 mg/m2 on days 1 and 8) was initiated [8], and the treatment was discontinued on day 14 due to various adverse events. Hematological toxicities of the National Cancer Institute Common Terminology Criteria for Adverse Events (NCI CTCAE, version 4.0) grade3 thrombocytopenia and neutropenia were noted on day 15. Non-hematological toxicities, depressed consciousness (Grade 1) and fever (Grade 2) were observed. CT and magnetic resonance imaging (MRI) examination did not suggest organic intracranial lesions, and the symptoms improved in 4 days. Lung congestion, respiratory distress, and hypoxemia (Grade 3) appeared on day 23. Echocardiography showed preserved cardiac function, and the brain natriuretic peptide (BNP) concentration was normal. No evidence of infectious diseases was detected. The other toxicities included syndrome of inappropriate secretion of antidiuretic hormone (SIADH) (Grade 3), increased ALP and γGTP (Grade 3), increased lipase (Grade 3), and hypothyroidism (Grade 2). All toxicities improved on day 28. In this case, hand-foot syndrome and stomatitis were not observed. In terms of efficacy, CT examination on day 20 showed a significant decrease in size of the lung metastases (Fig. 1b). The response of the lung metastases of RCC and no significant change for BDC were confirmed on day 35. She had subsequent temsirolimus monotherapy from January 2012 and it achieved tumor control for about 12 months. She is now under best supportive care 3.5 years after the initial chemotherapy.

### Pharmacokinetic sampling

The plasma levels of sunitinib and SU12662 were analyzed on days 13, 17, 21, 23, 27, and 33 using the high-performance liquid chromatography technique [9]. Peripheral blood was obtained before taking sunitinib on days 13, 17, 21, 23, 27 and 33. Then, samples (0.5 ml) were collected in tubes containing ethylenediamine tetraacetic acid (EDTA). Samples were centrifuged at 3500 rpm at 4 °C for 10 min, and 0.1 N NaOH was added to the supernatants. The compounds were extracted into 3 ml *t*-butyl methyl ether (TBME). After agitation for 5 min, the TBME phase was aspirated and evaporated to dryness (N_2_). Aliquots were subjected to high-performance liquid chromatography.

### High-performance liquid chromatography

The chromatographic system consisted of a mobile phase of a mixture [0.05 M phosphoric buffer (pH 3), acetonitrile, and B-7 low UV regent (Waters, Milford, MA, USA) at a ratio of 695:300:5] with an ODS column pumped at a flow rate of 0.3 ml/min and UV/VIS detection at 431 nm (0–12 min) and 250 nm (12–20 min). Sunitinib and SU12662 were purchased from TRC (Toronto Research Chemicals, Ontario, Canada). The internal standard was 4-methyl-mexirethyn [9]. The retention times for SU12662, sunitinib and the internal control were 5.8, 8.3 and 14.8 min, respectively.

### DNA sample preparation

The patient gave written, informed consent to participate in the present study. Genomic DNAs were extracted from whole blood (500 μL) by the SMITEST EX-R&D Nucleic Acid Extraction Kit (MBL Co., LTD. Nagoya, Japan). The concentration of the DNA was adjusted to 50 ng/μL.

### Genotyping of SNPs

ABCG2 polymorphism (−15662C/T) was genotyped using genomic polymerase chain reaction (PCR) and direct sequencing. Five μL of human genomic DNA (10 ng/μL), 20 μL of amplification reaction mixture, and 0.625 units Taq DNA polymerase were placed in reaction tubes. Amplification of the reaction mixture was carried out in 50 mM KCl, 10 mM Tris–HCl (pH 8.3), 1.5 mM MgCl_2_, 2 % dimethyl sulfoxide (DMSO), and 0.2 mM dNTPs, 0.2uM each of primers (FP: 5’-ACCCTGTCTGTCTCTACTAA-3’, RP: 5’-GTGATTACATTAAATGAGGTC-3’). PCR reactions were performed for 40 cycles, with denaturation at 94 °C for 30 s, annealing at 56 °C for 30 s, and extension at 72 °C for 30 s using a GeneAmp PCR System 9700 (Life Technologies). The sequence reaction was run in the ABI 3700 DNA analyzer (sequencing primer: 5’-CAACTCTCACCTATGAGTGA-3’) and analyzed using Sequencer computer software (Gene Codes Corporation, Ann Arbor, MI). Other polymorphisms, NR1I3 (5719C/T, 7738A/C, 7837 T/G), CYP1A1 (2455A/G), ABCG2 (1143C/T, 34G/A, 421C/A), ABCB1 (3435C/T, 1236C/T, 2677G/T), VEGFR2 (1191C/T) and FLT3 (738 T/C), were genotyped using the Illumina Human OmniExpress-12 BeadChip (Illumina Inc., San Diego, CA). A total of 200 ng of DNA (4 μL at 50 ng/μL) for the sample was processed according to the Illumina Infinium HD Assay Ultra protocol. BeadChip was imaged on the Illumina iScan System with iScan Control Software (v3.3.28). Normalization of raw image intensity data, genotype clustering, and individual sample genotype calls were performed using the Illumina GenomeStudio software (v2011.1), Genotyping Module (v1.9.4).

## Results

**Fig. 2:**
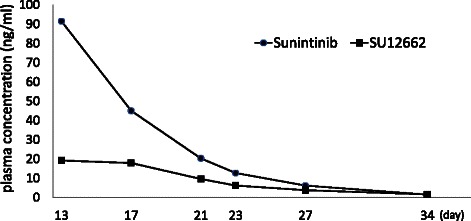
Plasma concentrations of Sunitinib (solid circle) and SU12662 (solid square) are shown. Horizontal axis indicates days after initiation of sunitinib administration

After administration of 25 mg/day of sunitinib from day 2 to day 13, the plasma trough concentrations were 91.5 ng/mL, decreasing to half on day 17. The plasma trough concentration of SU12662 was 19.2 ng/mL on day 13, and it also decreased on day 17 (Fig. 2). The patient’s condition gradually recovered following the decreases in the plasma levels. This suggests that the high plasma levels of sunitinib and SU12662 were possibly responsible for both the efficacy and toxicities observed in this case.

Gene polymorphisms that have been reported to be candidate SNPs related to the exposure and metabolism of sunitinib were analyzed (Table 1). In this case, TC in ABCB1 3435C/T, TC in 1236C/T and TT in 2677G/T were detected, and then there were two possibilities: TTT haplotype and TCT haplotype. CC in ABCG2 type, CC in -15622C/T, and CC in C421A type were also detected.

**Table 1:** SNPs and the genotypes in 13 previously reported genes

Gene	SNPs	rs Number	Genotype
NR1I3	5719C/T	rs2307424	TC
NR1I3	7738A/C	rs2307418	AA
NR1I3	7837 T/G	rs4073054	TG
CYP1A1	2455A/G	rs1048943	AA
ABCG2	1143C/T	rs2622604	CC
ABCG2	−15622C/T	rs55930652	CC
ABCG2	34G/A	rs2231137	GG
ABCG2	421C/A	rs2231142	CC
ABCB1	3435C/T	rs1045642	TC
ABCB1	1236C/T	rs1128503	TC
ABCB1	2677C/T	rs2032582	TT
VEGFR2	1191C/T	rs2305948	CC
FLT3	738 T/C	rs1933437	TC

## Discussion

Synchronous primary BDC was diagnosed during the course of metastatic RCC in this case. Although cytological and histological confirmation for BDC was not obtained, a series of radiological examinations showed typical findings of primary BDC rather than mRCC. RCCs due to hereditary cancer syndromes including Von Hippel-Lindau disease [10] and Birt-Hogg-Dube syndrome [11] are known to be associated with colon, breast, and ovarian cancers. On the other hand, BDC has been reported to be intercurrent with primary hepatocellular carcinoma [12] However, the synchronous occurrence of RCC and BDC is extremely rare, and the genetic backgrounds are unclear. To the best of our knowledge, this is the first report of systemic chemotherapy for a patient with concomitant RCC and BDC.

Combination therapy with sunitinib and gemcitabine, used in this case, was examined in a phase I trial for patients with advanced solid tumors [13]. Patients were given 25–37.5 mg/body daily of sunitinib, and 800 mg/m^2^ on days 1, 8 and 15 or 675 mg/m^2^ on days 1 and 8 of gemcitabine. In all patients groups, grade 3/4 hematological toxicities were observed for neutropenia (54 %), febrile neutropenia (6 %), thrombocytopenia (18 %), and anemia (12 %). Michaelson et al. assessed two different schedules of the combination therapies of (a) sunitinib on a 4-weeks-on-2-weeks-off schedule (Schedule 4/2) plus gemcitabine on days 1, 8, 22 and 29, and (b) sunitinib on a 2-weeks-on-1-week-off schedule (Schedule 2/1) plus gemcitabine on days 1 and 8 [14]. In three patients on Schedule 2/1 with 37.5 mg of sunitinib and 750 mg/m^2^ of gemcitabine, two patients showed grade 3 neutropenia and lymphopenia, and one patient had grade 3 leukopenia. Increments of the doses of both drugs induced grade 4 hematological and non-hematological toxicities, but no significant drug-drug interaction was suggested.

A case of pancreatic cancer and RCC that was treated with combination chemotherapy with 37.5 mg of sunitinib (4-weeks-on-2-weeks-off) and 750 mg/m^2^ of gemcitabine on day 1, 8 and 15 was also reported. Two courses of the therapy achieved high relative dose intensity and partial responses. Although the dose of gemcitabine was decreased because of neutropenia, the therapy was safely continued for 25 weeks [15].

Comparing these reports, the present case experienced relatively intense and various adverse events even though lower doses of drugs were administered. Since hematological toxicities and liver toxicity were also observed in the previous reports, they might be caused by the combination therapy. On another front, hypothyroidism was thought to be induced by sunitinib and pulmonary edema might be caused by the acceleration of vascular endothelial growth factor (VEGF) via sunitinib.

One of the possible reasons for the enhancement of adverse events was suggested to be the elevated plasma concentration of sunitinib. A positive correlation between plasma concentration of sunitinib and adverse events was reported by Houk [16]. The previous phase I trial also showed that sunitinib-induced toxicities appeared in a dose-dependent manner [13]. Plasma trough concentrations of sunitinib and SU12662 were 91.5 ng/mL and 19.2 ng/mL, respectively, on day 13, when the trough concentrations might be in a steady-state. Daily administration of sunitinib alone in Japanese patients with pancreatic endocrine tumors showed mean dose-corrected (reference dose: 37.5 mg) C_trough_ values that were within the range of 41.7-53.9 ng/mL for sunitinib, 19.6-25.7 ng/mL for SU12662, and 62.9-77.5 ng/mL for total drug [17]. In terms of PK analysis of the combination of sunitinib and gemcitabine, the Cmax of sunitinib on day 8 of 50 mg daily administration was 71.5 ng/mL (CV% 58) (median 60.0) and that of SU12662 was 27.5 ng/mL (CV% 68) (median 20.9) [14]. Although direct comparison between the values of Cmax and trough concentration is difficult, and data on the trough concentration in combination therapy are not available, the plasma trough concentrations of sunitinib and SU12662 in the present case were relatively high.

The plasma concentration of sunitinib is regulated by various factors, including drug-transporters in epithelial cells of the gastrointestinal tract for absorption of the drug. Sunitinib was thought to be a substrate for ATP-binding transporters ABCG2 and ABCB1 [18–20]. It has been reported that the TTT haplotype of ABCB1 was generally associated with decreased expression of ABCB1 and subsequent higher-exposure of its substrates [21, 22]. In addition, van Erp et al. reported that TTT haplotype was associated with the risk of hand-foot syndrome with sunitinib [23]. However, Garcia-Donas demonstrated that the ABCB1 polymorphisms were not significantly associated with the efficacy and toxicities of sunitinib [24]. While the ABCB1 haplotype of the present case was possibly TTT type, hand-foot syndrome did not appear. Therefore, the relationships between ABCB1 polymorphism and the high plasma concentrations of sunitinib and SU12662 were not clarified. Sunitinib has also been reported to be a substrate for ABCG2, and its polymorphism of 421C/A was correlated with increased exposure of sunitinib [25]. In addition, TT haplotypes of ABCG2 -15622C/T, and 1143C/T showed increased risks of adverse events [23]. These series of ABCG2 polymorphisms were not found in the present case.

The drug-metabolizing enzyme CYP3A4 is a key enzyme in sunitinib metabolism, and NR1I3 is one of the regulating factors of CYP3A4 expression [26]. CYP1A1 is known to be associated with the metabolism of tyrosine kinase inhibitors. Since these might possibly affect the plasma concentration of sunitinib, polymorphisms of these factors were also examined in this case, but no significant correlation was found. Therefore, polymorphisms of specific genes, which might be closely associated with the PK of sunitinib, could not be eliminated as possible reasons for the high exposure of sunitinib and various adverse events; other factors should be considered.

In the present study, PK analysis of gemcitabine could not be performed. However, to the best of our knowledge, gemcitabine does not induce or inhibit CYP450, and it is not a substrate of ABCB1 and ABCG2. CYP3A4 has not been known to be involved in gemcitabine metabolism [14]. Therefore, it is less likely that combination use of gemcitabine might affect the plasma concentration of sunitinib.

After submission of this manuscript, several clinical studies employed the combination of sunitinib and gemcitabine had been published. A randomized phase II study in advanced pancreatic cancer demonstrated the combination regimen could not show sufficient superior efficacy compared to gemcitabine monotherapy but was associated with more toxicity [27]. Triplet regimens consisting of the combination and cisplatin or capecitabine in advanced solid tumors exhibited prominent toxicities suggesting difficulties of further developments of triplet regimens [28, 29].

## Conclusions

An extremely rare case of the concomitant occurrence of RCC and BDC was treated by combination therapy with sunitinib and gemcitabine, and clinical response was achieved. Various adverse events might be associated with increased plasma concentrations of sunitinib. Possible mechanisms of high exposure of sunitinib might include gene polymorphisms of drug-transporters, but unknown mechanisms induced by the combination use of two drugs should be investigated. Combination chemotherapy against double cancers requires more careful management, and it is important to identify adequate biomarkers for predicting efficacy and toxicities.

## Consent

Written informed consent was obtained from the patient for publication of this Case report and any accompanying images. A copy of the written consent is available for review by the Series Editor of this journal.

## Abbreviations

mRCC: Metastatic renal cell carcinoma
BDC: Bile duct cancer
SNPs: Single nucleotide polymorphisms
RCC: Renal cell carcinoma
VEGFR: Vascular endothelial growth factor receptor
PDGFR: Platelet-derived growth factor receptor
PK: Pharmacokinetics
PD: Pharmacodynamics
CT: Computed tomography
NCI CTCAE: National Cancer Institute Common Terminology Criteria for Adverse Events
MRI: Magnetic resonance imaging
BNP: Brain natriuretic peptide
SIADH: Syndrome of inappropriate secretion of antidiuretic hormone
EDTA: Ethylenediamine tetraacetic acid
TBME: *t*-butyl methyl ether
PCR: Polymerase chain reaction
VEGF: Vascular endothelial growth factor
